# Relationship between the severity of pre-frailty and the degree of adaptation of Ninjin’yoeito (NYT) on pre-frailty

**DOI:** 10.3389/fragi.2024.1304217

**Published:** 2024-04-12

**Authors:** Haruka Amitani, Hajime Suzuki, Hironori Kobayashi, Masaru Murayama, Nanami Sameshima Uto, Eishi Kuroda, Yoshiki Kobayashi, Momoko Kawabe, Marie Amitani, Akio Inui, Yoshinori Marunaka

**Affiliations:** ^1^ Pharmacological Department of Herbal Medicine, Kagoshima University Graduate School of Medical and Dental Sciences, Kagoshima, Japan; ^2^ Division of Psychosomatic Internal Medicine, Kagoshima University Graduate School of Medical and Dental Sciences, Kagoshima, Japan; ^3^ Division of Oral and Maxillofacial Surgery, Kagoshima University Graduate School of Medical and Dental Sciences, Kagoshima, Japan; ^4^ Medical Research Institute, Kyoto Industrial Health Association, Kyoto, Japan; ^5^ Kampo Research Laboratories, Kracie Pharma, Ltd, Tokyo, Japan; ^6^ Education Center for Doctors in Remote Islands and Rural Areas, Kagoshima University Graduate School of Medical and Dental Sciences, Kagoshima, Japan; ^7^ Division of Community-Based Medicine, Kagoshima University Graduate School of Medical and Dental Sciences, Kagoshima, Japan; ^8^ Research Organization of Science and Technology, Ritsumeikan University, Kusatsu, Japan

**Keywords:** frailty, Ninjin’yoeito (NYT), Revised Japanese version of the cardiovascular health study (revised J-CHS), Kihon checklist (KCL), general health questionnaire 12 (GHQ12)

## Abstract

With the global trend towards longer life expectancies, there’s an increasing emphasis on not just living longer, but also maintaining health and wellbeing into older age. This study explores the efficacy of Ninjin’yoeito (NYT) in the early stages of frailty, a critical period for preventive interventions. Taking account of the knowledge gap regarding the association between early frailty and NYT, we use data from workplace health checkups to examine the relationship between pre-frailty severity and NYT adaption. The objective of our research is to enhance the comprehension of early treatments using NYT to prevent the progression of frailty. A total of 314 employees of the Kyoto Industrial Health Association who received workplace health checkups between November 2021 and March 2023 and consented to this study were included in the analysis. Information on gender, age, body mass index (BMI), NYT-specific symptoms assessment, the Japanese version of the General Health Questionnaire-12 (GHQ-12), and the Kihon Checklist (KCL) were obtained. The correlation analysis revealed that there was a strong positive correlation between the number of applicable NYT indications and the GHQ-12 score (r = 0.5992, *p* < 0.0001). Similarly, a moderate positive correlation was observed between the number of applicable NYT indications and the KCL score (r = 0.5030, *p* < 0.0001). In the multivariate analysis, both GHQ-12 (β = 0.49, SE = 0.06, t = 7.66, 95% CI: 0.36 to 0.62, *p* = 0.000) and KCL (β = 0.54, SE = 0.12, t = 4.29, 95% CI: 0.29 to 0.79, *p* = 0.000) showed significant positive associations with the variance in the number of applicable NYT indications, indicating that higher scores on these measures were related to a greater number of indications. NYT has the potential to be utilized not only as a therapeutic intervention for frailty, but also as a preventive measure.

## Highlights

► This is the first study to investigate the relationship between the severity of frailty and the degree of adaption to Ninjin’yoeito (NYT) in employees who have underwent a workplace health checkup.
► A number of recent articles have documented the effectiveness of NYT in addressing frailty.
► We selected NYT-specific symptoms assessment, the Japanese version of the General Health Questionnaire-12 (GHQ-12), and the Kihon Checklist (KCL) to conduct our investigation.

## Introduction

Advances in modern medicine since the 1950s have contributed significantly to extending human life expectancy, leading to a significant increase in the number and proportion of the elderly in the world population (Rau et al., 2008). A recent United Nations report underscores this demographic shift, projecting that the number of people aged 65 and over will more than double from 727 million in 2020 to more than 1.5 billion by 2050 (United Nations, 2023). This demographic trend underscores the importance of extending “healthy life expectancy. Healthy life expectancy is the period during which an individual can maintain daily life without health constraints. Addressing this will require not only medical advances, but also preventive measures to ensure that individuals can lead active and healthy lives into old age, thereby reducing healthcare costs and increasing overall wellbeing.

Frailty has emerged as a major obstacle in maintaining the health and extending the healthy life expectancy of elderly individuals. Frailty is a condition characterized by heightened physiological fragility, which has been linked to elevated chances of adverse health outcomes such as morbidity, falls, hospitalization, long-term care, institutionalization, and mortality. These consequences impose significant demands on healthcare and social systems (Clegg et al., 2013; Chen et al., 2014; Cesari et al., 2016). The worldwide population of aged individuals is steadily rising, leading to an increased recognition of the need of preventing and delaying the onset of frailty (Cesari et al., 2016; Puts et al., 2017).

The primary objective of the major statutory health checkup and advice programs in Japan is to mitigate the risk of lifestyle diseases by encouraging the adoption of healthy habits through health monitoring initiatives (Higa et al., 2021). In Japan, it is well acknowledged that the mere provision of checkup results to individuals is inadequate in eliciting behavioral modifications. These programs mandate companies to give health screenings for their employees, as well as further support like as medical consultations and health counseling, in cases where the checkup findings indicate a need for such assistance. In other words, the workplace health checkups conducted in Japan allow to get a comprehensive set of health-related information pertaining to employees, collected concurrently.

Ninjin’yoeito (NYT), a conventional Japanese Kampo medicine, is designed for individuals with a weakened constitution due to aging, illness recovery, diminished appetite, cold extremities, anemia, and nocturnal sweating (Uto et al., 2018). Containing twelve potent herbs such as ginseng, Japanese angelica root, peony root, rehmannia root, atractylodes rhizome, poria sclerotium, cinnamon bark, astragalus root, citrus Unshiu peel, polygala root, schisandra fruit, and glycyrrhiza, NYT is noted for its broad-spectrum efficacy in revitalizing the body’s constitution, alleviating fatigue, malaise, anorexia, insomnia, and bolstering physical strength post-recovery (Miyano et al., 2018; Uto et al., 2018; Suzuki et al., 2019; Takayama et al., 2019; Hirai et al., 2020). Its application in modern clinical settings, particularly for frailty management in gastrointestinal, respiratory, and urinary functions, demonstrates its adaptability and relevance in contemporary healthcare. The effectiveness of NYT in addressing frailty has been documented in recent articles (Miyano et al., 2018; Uto et al., 2018; Suzuki et al., 2019; Takayama et al., 2019; Hirai et al., 2020), with an increasing understanding of its underlying mechanisms (Zhang et al., 2021; Amitani et al., 2022), though these are still under active investigation. This study aims to explore the relationship between frailty severity and the adaptation to NYT, utilizing data from workplace health checkups to deepen our understanding of its therapeutic potential and the applicability of NYT indications to prevent the progression of frailty.

## Materials and methods

### Study design and participants

This research is a multi-institutional collaboration between the Department of Kampo Pharmacology, Graduate School of Medical and Dental Sciences, Kagoshima University, Kyoto Industrial Health Association Foundation, and Gene Quest Co. The study was approved by the Ethical Review Committee of Kagoshima University with the committee’s reference number 210176 (G449) and the Ethical Review Committee of Kyoto Industrial Health Association (approval number: S19-0008-02). Employees of the Kyoto Industrial Health Association who received workplace health checkups between November 2021 and March 2023 and consented to this study were included in the analysis.

### Data collection

Information on gender, age, body mass index (BMI), NYT-specific symptoms assessment, the Japanese version of the General Health Questionnaire-12 (GHQ-12), and the Kihon Checklist (KCL) were obtained.

In this study, we used a questionnaire specifically designed to assess indications for NYT, as shown in Table 1. Participants reported their experience during the past week of six main symptoms: loss of energy, general malaise, anorexia, night sweats, cold hands and feet, and anemia. Participants recorded the presence or absence of these symptoms daily using a scale from 0 (days without symptoms) to 7 (symptoms present daily). For the analysis, these daily reports were tabulated to produce an individual score of 0–7 for each symptom and a cumulative score across all symptoms. This comprehensive scoring system allowed for a nuanced assessment of the extent to which participants’ experiences matched NYT-adaptive symptoms and provided a basis for analyzing the correlation between the severity of frailty and NYT-adaptability based on self-reported health indicators.

**TABLE 1:** NYT-specific symptoms assessment.

Question	Score
Physical weakness: How many days in the past week did you feel?	0–7 (days)
General fatigue: How many days in the past week did you feel?	0–7 (days)
Loss of appetite: How many days in the past week did you feel?	0–7 (days)
Night sweats: How many days in the past week did you feel?	0–7 (days)
Coldness: How many days in the past week did you feel?	0–7 (days)
Anemia: How many days in the past week did you feel?	0–7 (days)
Total	0–42

NYT: Ninjin’yoeito

In response to the questions regarding the assessment of anemia, this questionnaire was designed to capture subjective experiences of anemia-related symptoms rather than clinical diagnoses. Participants were asked to reflect on their experiences over the past week and report the number of days they experienced anemia-related symptoms. This approach was intended to measure subjective perceptions of anemia-related symptoms and their impact on daily life, consistent with the holistic assessment principles of Kampo medicine. This self-reported questionnaire was part of a broader set of indicators used to understand potential indications for NYT and was not intended as a stand-alone diagnostic tool for anemia.

The GHQ-12 questionnaire is a widely used tool for screening psychological distress and mental health (Goldberg and Williams, 1988). It consists of 12 questions that assess an individual’s recent experiences and feelings. Each question typically offers response options, such as “more than usual” or “less than usual,” allowing individuals to indicate their level of agreement or disagreement. Each item assesses the severity of a mental problem over the past few weeks using a 4-point Likert-type scale (from 1 to 4). The score was used to generate a total score ranging from 0 to 48. The total score provides an indication of psychological wellbeing, with higher scores suggesting higher levels of distress. The validity and reliability of the GHQ-12 have been extensively investigated and confirmed in studies, including that conducted in 15 centers worldwide (Goldberg et al., 1997). Previous studies suggest that adolescents interpret the GHQ-12 in a manner similar to adults (Banks, 1983; Tait et al., 2003; Baksheev et al., 2011). The validity and reliability of the Japanese version of the GHQ-12 have been confirmed in adolescents (Nakagawa, 1982). The total score of GHQ-12 was used for the analysis.

The Kihon Checklist (KCL) is a concise and precise tool developed by the Ministry of Health Labor and Welfare in Japan used to assess the functional decline and frailty in older adults (Satake et al., 2016). It consists of 25 questions covering various domains, including physical strength, nutrition, oral health, social relationships, and cognitive function. Each question is answered with either a “yes” or “no” response. The total score ranges from 0 to 25, with a higher score indicating a higher level of frailty or functional decline (Satake et al., 2016). Total KCL scores were used in the analysis. In addition, according to the total score, the patients were divided into three groups according to the cutoff values: frail, pre-frail, and robust (Satake et al., 2016).

### Statistical analysis

All statistical analyses were used by Stata version 16 (StataCorp LLC, College Station, TX, USA) and GraphPad Prism version 9.5.1 for MacOS (GraphPad Software, San Diego, CA, USA). The threshold of significance was set at 0.05. Descriptive statistics were calculated for all variables included in the study. We tested for normality using the Shapiro-Wilk test. Correlation analysis was performed to examine the relationships between the number of applicable NYT indications and the GHQ-12 score and between the number of applicable NYT indications and the KCL score. Spearman r was calculated to determine the strength and direction of the associations. A one-way analysis of variance was performed from the KCL score on the comparison of the number of applicable NYT indications in the three groups of frail, pre-frail, and robust. The multiple regression analysis was conducted to examine the relationship between the number of applicable NYT indications (dependent variable) and the KCL total score and GHQ12 total score (independent variables), while controlling for age, gender, and BMI as covariates. A multivariate regression analysis was also conducted on the association of GHQ-12 and KCL scores with each of the NYT indications, adjusted for age, sex, and BMI.

## Results

The baseline demographic information is shown in Table 2. The sample consisted of 314 participants, with 126 males and 188 females. The median age of the participants was 47.0 years [interquartile range: 41.7–54.0]. The median BMI was 22.0 kg/m^2^ [19.7–24.2]. The mean number of NYT applicable indications for males was 3 [0–7.2], while for females it was 6 [1–12]. The median GHQ-12 score for males was 25.0 [21.0–30.0], and for females, it was 25.0 [23.0–29.0]. Additionally, the KCL median score for males was 7 [5–9], and for females, it was 6 [4–8]. None of them reported depression or other psychiatric disorders in their medical history. We tested for normality using the Shapiro-Wilk test and found that only the GHQ-12 total score for males was normally distributed.

**TABLE 2:** Participants’ characteristics: results expressed as median [interquartile range] or count (percentage).

	Total (n = 314)	Male (n = 126)	Female (n = 188)
Age (years)	47.0 [41.7–54.0]	52.0 [43.7–58.0]	46.0 [41.0–50.0]
BMI (kg/m^2^)	22.0 [19.7–24.2]	22.3 [21.9–22.6]	20.7 [18.9–22.8]
Number of NYT applicable	4 [1–10]	3 [0–7.2]	6 [1–12]
GHQ-12	25.0 [22.0–29.0]	25.0 [21.0–30.0]	25.0 [23.0–29.0]
KCL	6 [5–8]	7 [5–9]	6 [4–8]

Abbreviations:BMI: body mass index; NYT: Ninjin’yoeito; GHQ: general health questionnaire; KCL: kihon check list.

**FIGURE 1:**
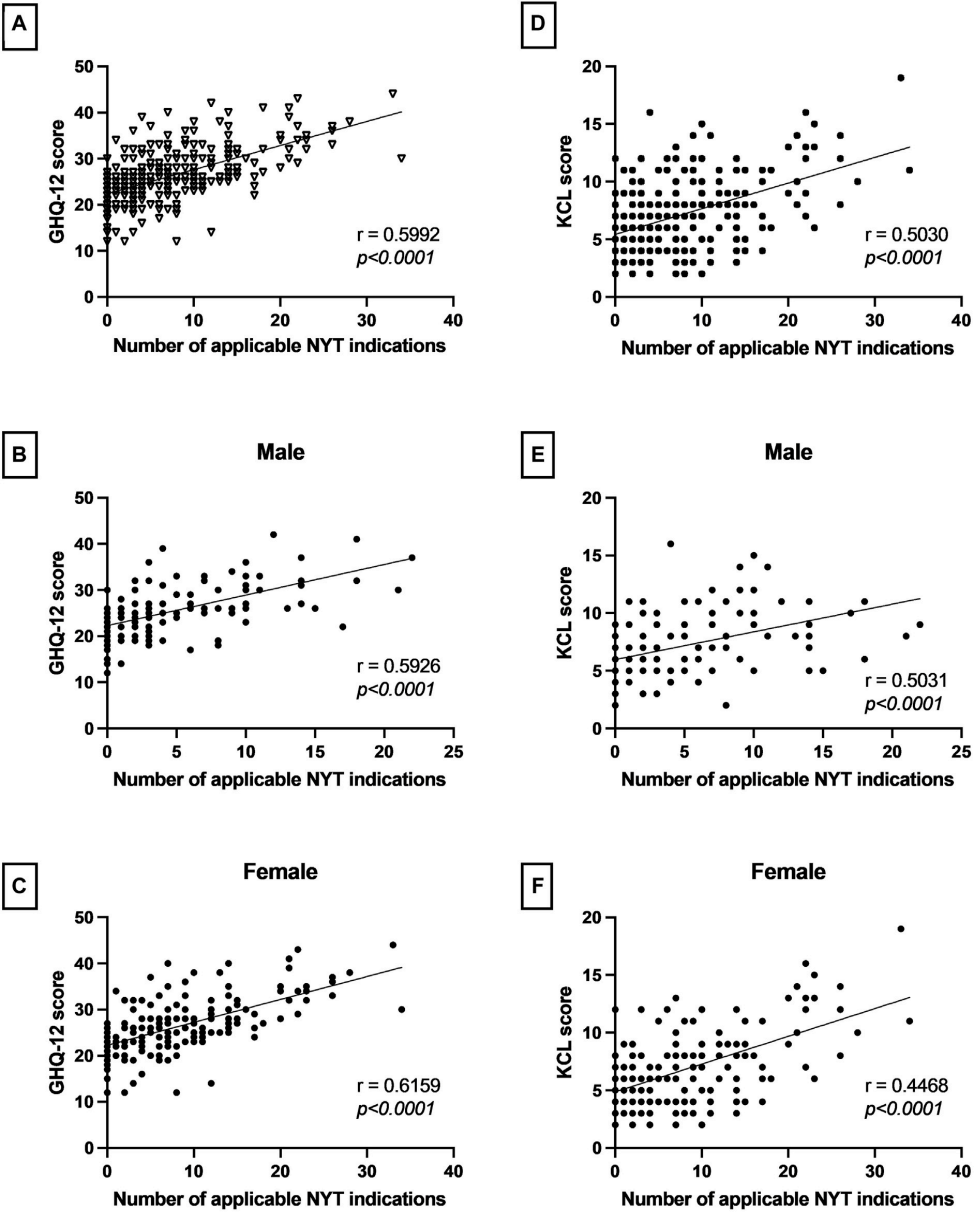
The correlations between the number of Ninjin’yoeito (NYT) adaptations and rating scores, both overall and by gender. **(A–C)** The correlation analysis performed in this study showed a significant positive correlation between the number of NYT indications and GHQ-12 scores, both overall and by gender. **(D–F)** Significant positive correlations were also observed between the number of NYT indications and KCL scores, both overall and by gender. These results highlight the significant relationship between the number of applicable NYT indications and both psychological distress and severity of symptom frailty.

**FIGURE 2:**
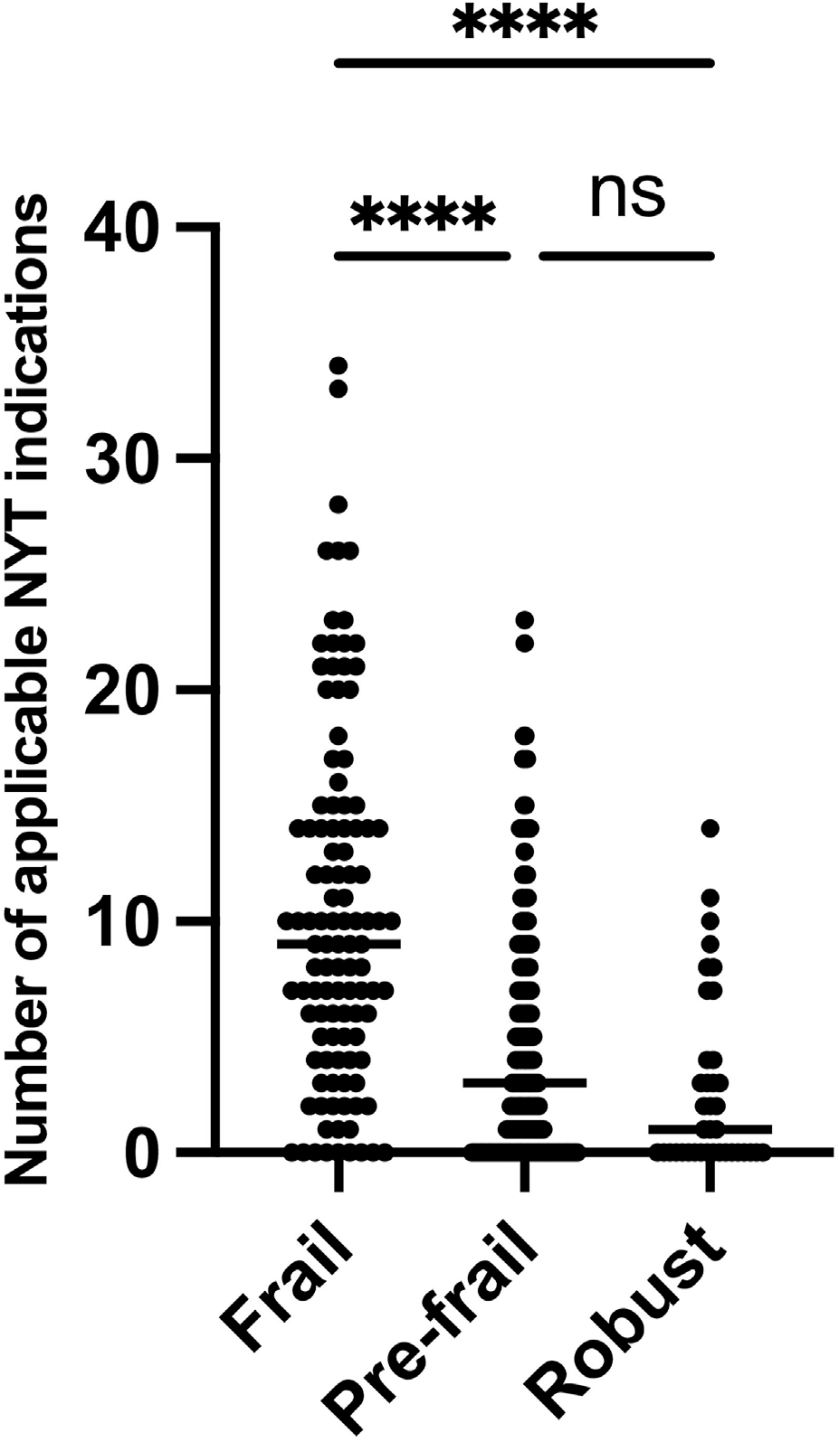
Comparative analysis of NYT scores for frail, prefrail, and robust groups. NYT: Ninjin’yoeito.

As shown in Figure 1, the correlation analysis revealed that there was a strong positive correlation between the number of applicable NYT indications and the GHQ-12 score (r = 0.5953, *p* < 0.0001). Similarly, a moderate positive correlation was observed between the number of applicable NYT indications and the KCL score (r = 0.4289, *p* < 0.0001). When examined by gender, mild to moderate correlations were found. Figure 2 shows that the number of NYT scores was significantly higher in the frailty group than in the pre-frailty and robust groups (*p* < 0.0001).

Multiple regression analysis was conducted to examine the relationship between the number of applicable NYT indications and the independent variables (KCL total score and GHQ12 total score), while controlling for age, gender, and BMI. In the multivariate analysis, both GHQ-12 (β = 0.49, SE = 0.06, t = 7.66, 95% CI: 0.36 to 0.62, *p* = 0.000) and KCL (β = 0.54, SE = 0.12, t = 4.29, 95% CI: 0.29 to 0.79, *p* = 0.000) showed significant positive associations with the variance in the number of applicable NYT indications, indicating that higher scores on these measures were related to a greater number of indications (Table 3A). A multivariate analysis was also conducted on the association of GHQ-12 and KCL scores with each of the NYT measures. The results showed that GHQ-12 scores were significantly associated with physical weakness, general fatigue, and loss of appetite (Table 3B). In addition, KCL scores were significantly associated with anemia in addition to physical weakness, general fatigue, and loss of appetite (Table 3C).

**TABLE 3:** (A) Multiple regression analysis of the relationship between the dependent variable Ninjin’yoeito indications and each independent variable, adjusted for age, sex, and BMI. (B) Multiple regression analysis of the relationship between the dependent variable GHQ-12 scores and each independent variable, adjusted for age, sex, and BMI. (C) Multiple regression analysis of the relationship between the dependent variable KCL scores and independent variables, adjusted for age, sex, and BMI.

(A)						
NYT	Univariate	Multivariate				
Variable	*p*	β	SE	t	95%CI	*p*
Age	0.004	−0.00	0.03	−0.02	−0.07 – 0.07	0.982
Sex	0.000	2.78	0.66	4.17	1.47 – 4.10	0.000
BMI	0.066	−0.01	0.01	−0.57	−0.05 – 0.02	0.570
GHQ-12	0.000	0.49	0.06	7.66	0.36 – 0.62	0.000
KCL	0.000	0.54	0.12	4.29	0.29 – 0.79	0.000

(B)						
GHQ-12	Univariate	Multivariate				
Variable	*p*	β	SE	t	95%CI	*p*
Age	0.005	−0.07	0.03	−2.15	−0.13 – 0.00	0.033
Sex	0.238	−0.65	0.61	−1.07	−1.85 – 0.54	0.287
BMI	0.546	−0.00	0.01	−0.28	−0.04 – 0.03	0.781
Physical weakness	0.000	0.66	0.12	4.21	0.35 – 0.97	0.000
General fatigue	0.000	0.84	0.17	4.92	0.50 – 1.18	0.000
Loss of appetite	0.000	0.85	0.24	3.42	0.36 – 1.34	0.001
Night sweats	0.000	0.36	0.19	1.87	−0.01 – 0.75	0.062
Coldness	0.000	0.11	0.11	1.01	−0.10 – 0.34	0.312
Anemia	0.001	0.07	0.26	0.27	−0.44 – 0.59	0.784

(C)						
KCL	Univariate	Multivariate				
Variable	*p*	β	SE	t	95%CI	*p*
Age	0.025	−0.04	0.01	−2.43	−0.07 – 0.00	0.016
Sex	0.319	−1.04	0.32	−3.19	−1.68 – 0.39	0.002
BMI	0.468	0.00	0.00	0.34	−0.01 – 0.02	0.733
Physical weakness	0.000	0.36	0.08	4.31	0.19 – 0.53	0.000
General fatigue	0.000	0.22	0.09	2.50	0.04 – 0.41	0.013
Loss of appetite	0.000	0.51	0.13	3.87	0.25 – 0.77	0.000
Night sweats	0.002	0.01	0.10	1.04	−0.09 – 0.31	0.299
Coldness	0.002	−0.01	0.06	−0.31	−0.13 – 0.10	0.755
Anemia	0.000	0.49	0.14	3.53	0.22 – 0.77	0.000

SE: standard error; 95%CI: 95% confidence interval; BMI: body mass index; NYT: Ninjin’yoeito; GHQ: general health questionnaire; KCL: kihon check list.

## Discussion

To the best of our current understanding, this is the first study to investigate the relationship between the severity of frailty and the degree of adaption to NYT in employees who have underwent a workplace health checkup. The findings of the study indicated a positive correlation between the number of applicable NYT indications and both the KCL total score and the GHQ12 total score.

The implementation of a long-term care insurance (LTCI) system in Japan has been prompted by the fast aging of the population (Tsutsui and Muramatsu, 2007). The LTCI is an insurance system designed to provide support to elderly individuals who are frail or disabled, helping them with their daily tasks. Individuals who are above the age of 65 and require assistance are eligible to receive formal care services, subject to the standards established by the government. The utilization of the Kihon Checklist (KCL) is recommended by the Ministry of Health, Labour and Welfare for the purpose of screening individuals within the target group who are in need of nursing care preventive and Long-Term Care Insurance (LTCI) services. The KCL was developed with the aim of identifying the many health concerns that arise from comprehensive geriatric symptoms. The tool is specifically designed to address the needs of the older population. Additionally, the KCL is utilized to assess the efficacy of interventions implemented (Tsutsui and Muramatsu, 2005; Satake et al., 2016; Sewo Sampaio et al., 2016; Ito et al., 2021). Frailty is frequently delineated by sarcopenia, a debilitating decline in muscle mass, strength, and functionality that also includes various physiological, psychological, and socio-environmental aspects (Rizzoli et al., 2013; Williams et al., 2019). The KCL also conducts assessments in these psychological, and socio-environmental aspects.

The frailty phenotype, as outlined by Fried et al., incorporates data derived from the Cardiovascular Health Study. This comprehensive definition encompasses several key indicators, including unintentional weight loss of 10 pounds within the previous year, self-reported exhaustion, weakness as measured by grip strength, slow walking speed, and low levels of physical activity (Fried et al., 2001). It is important to note that this definition of frailty is one of the most widely used definitions of frailty. Based on the frailty criteria established by Fried et al., the KCL has strong validity in evaluating frailty (Ogawa et al., 2011; Satake et al., 2016). The study indicated a positive correlation between the number of applicable NYT indications and the KCL total score. The findings of our investigation can be characterized as having unveiled a positive correlation between the number of NYT adaptations and the likelihood of frailty occurrence. Furthermore, the association of KCL scores with NYT application items such as anemia, low physical weakness, general fatigue, and loss of appetite, extends the applicability of the NYT; the KCL assesses a broader range of functional and health-related factors, indicating that the presence of anemia-related symptoms further complicates the complexity of frailty among individuals. The KCL is a multifaceted treatment that is designed to assess the effects of anemia on the health of the individual. This suggests that NYTs with a multifaceted treatment approach may be particularly beneficial in managing the comprehensive symptoms associated with frailty, addressing both physical and psychological aspects, and improving overall quality of life.

While the traditional association between frailty and older adults is widely recognized, an integrative review by Loecker et al. (2021) highlights the presence of frailty in individuals aged 18–65 years, highlighting the multidimensional nature of frailty and the importance of early intervention (Loecker et al., 2021). Complementing this, the UK Biobank study by Hanlon et al. (2018) shows an association between frailty and pre-frailty in a middle-aged population and further advocates preventive measures well before conventional old age (Hanlon et al., 2018). Taken together, these studies support a broader understanding of frailty beyond old age and highlight the potential for early intervention to alter the trajectory of frailty positively. The study’s focus on a younger cohort (mean age 47.7 years) is in line with this evolving perspective and is intended to contribute to the basic knowledge about early frailty markers and inform strategies for lifelong frailty prevention. Although the findings in this study relate to a younger cohort, they provide valuable insight into the frailty reserve that may precede more pronounced frailty in later years; direct application to the population aged 65 years and older may be limited by differences in comorbidity profiles and the effects of aging. However, the findings on early indicators in this study are useful for prevention strategies and are consistent with previous literature.

The GHQ-12 is a highly prevalent instrument utilized for evaluating quality of life. According to the previous study, the poor health status of persons exhibiting frailty was found to have a substantial impact on their capacity to participate in various activities (Puts et al., 2007). Consequently, the persons who exhibited frailty had a decline in their overall quality of life when compared to those who did not display frailty (Puts et al., 2007). The finding of our investigation indirectly correspond with the results documented in the previous study. Moreover, it was shown that there exists a positive correlation between the number of applicable NYT indications and the extent of impairment in one’s quality of life. This correlation suggests that those who experience more of these symptoms that could potentially be treated with NYT report higher quality of life impairment. This finding highlights the potential for NYT not only to address specific symptoms, but also to improve the overall quality of life of those who exhibit these symptoms. Multivariate analysis revealed a significant relationship between subjective health ratings and the specific symptoms NYT is trying to address: there was a significant association between GHQ-12 scores and symptoms such as low physical weakness, general fatigue, and loss of appetite, suggesting that those experiencing high psychological distress, as measured by the GHQ-12 suggesting that those experiencing high psychological distress as measured by the GHQ-12 were more likely to report these specific physical symptoms. This concordance underscores the interconnectedness of psychological wellbeing and physical health and indicates the potential for the NYT to address both domains simultaneously.

This study has several limitations. First, we used questionnaire-based assessments about physical activity; however, objective assessments such as pedometers and wearable devices should also be considered. Second, this study did not verify the impact of the number of medications taken on frailty. Polypharmacy has been reported to be a risk factor for frailty (Gutierrez-Valencia et al., 2018). Third, this observational study has provided evidence on the correlation between the degree of adaption to NYT and the severity of frailty in employees. However, it is important to note that the study does not establish a causal relationship between these variables. Further investigation is required to clarify the cause-and-effect connection by means of intervention trials conducted on individuals diagnosed with frailty. Finally, the interpretation issues associated with this study were caused by the fact that the population was younger. However, we recognize that this approach has limitations, including a potential gap in direct applicability to persons beyond 65, but we think our findings contribute significantly to our understanding of early frailty indications and preventative efforts.

The deterioration of frailty in elderly individuals is expected to intensify (Lee et al., 2014; Trevisan et al., 2017). Furthermore, there have been reports indicating that it can elevate the likelihood of experiencing negative health consequences, such as long-term care requirements and fatality (Cawthon et al., 2007; Nielsen et al., 2021). However, it should be noted that frailty is a condition that may be reversed and is subject to change throughout time, with the possibility of both improvement and deterioration (Shinkai et al., 2016). There is an expectation that forthcoming breakthroughs would result in further improvements in preventative strategies and appropriate treatments aimed at addressing frailty, a complex condition influenced by several factors.

## Conclusion

The findings of our study suggest that NYT has the capacity to offer advantages even in cases of mild and moderate frailty. In a broader sense, NYT has the potential to be utilized not only as a therapeutic intervention for frailty, but also as a form of prevention.

## Data Availability

The original contributions presented in the study are included in the article/Supplementary material, further inquiries can be directed to the corresponding author.

## References

[B1] Amitani H. Chiba S. Amitani M. Michihara S. Takemoto R. Han L. (2022). Impact of Ninjin’yoeito on frailty and short life in klotho-hypomorphic (Kl/Kl) mice. Front. Pharmacol. 13, 973897. Epub 2022/11/11. 10.3389/fphar.2022.973897 36353482 PMC9637981

[B2] Baksheev G. N. Robinson J. Cosgrave E. M. Baker K. Yung A. R. (2011). Validity of the 12-item general health questionnaire (Ghq-12) in detecting depressive and anxiety disorders among high School students. Psychiatry Res. 187, 291–296. Epub 2010/11/12. 10.1016/j.psychres.2010.10.010 21067813

[B3] Banks M. H. (1983). Validation of the general health questionnaire in a young community sample. Psychol. Med. 13, 349–353. Epub 1983/05/01. 10.1017/s0033291700050972 6878521

[B4] Cawthon P. M. Marshall L. M. Michael Y. Dam T. T. Ensrud K. E. Barrett-Connor E. (2007). Frailty in older men: prevalence, progression, and relationship with mortality. J. Am. Geriatr. Soc. 55, 1216–1223. Epub 2007/07/31. 10.1111/j.1532-5415.2007.01259.x 17661960

[B5] Cesari M. Prince M. Thiyagarajan J. A. De Carvalho I. A. Bernabei R. Chan P. (2016). Frailty: an emerging public health priority. J. Am. Med. Dir. Assoc. 17, 188–192. 10.1016/j.jamda.2015.12.016 26805753

[B6] Chen X. Mao G. Leng S. X. (2014). Frailty syndrome: an overview. Clin. interventions aging 9, 433–441. 10.2147/CIA.S45300 PMC3964027 24672230

[B7] Clegg A. Young J. Iliffe S. Rikkert M. O. Rockwood K. (2013). Frailty in elderly people. lancet 381, 752–762. 10.1016/S0140-6736(12)62167-9 23395245 PMC4098658

[B8] Fried L. P. Tangen C. M. Walston J. Newman A. B. Hirsch C. Gottdiener J. (2001). Frailty in older adults: evidence for a phenotype. J. Gerontol. A Biol. Sci. Med. Sci. 56, M146–M156. Epub 2001/03/17. 10.1093/gerona/56.3.m146 11253156

[B9] Goldberg D. P. Gater R. Sartorius N. Ustun T. B. Piccinelli M. Gureje O. (1997). The validity of two versions of the ghq in the who study of mental illness in general health care. Psychol. Med. 27, 191–197. Epub 1997/01/01. 10.1017/s0033291796004242 9122299

[B10] Goldberg D. P. Williams P. (1988). A user’s guide to the general health questionnaire. (No Title).

[B11] Gutierrez-Valencia M. Izquierdo M. Cesari M. Casas-Herrero A. Inzitari M. Martinez-Velilla N. (2018). The relationship between frailty and polypharmacy in older people: a systematic review. Br. J. Clin. Pharmacol. 84, 1432–1444. Epub 2018/03/27. 10.1111/bcp.13590 29575094 PMC6005607

[B12] Hanlon P. Nicholl B. I. Jani B. D. Lee D. McQueenie R. Mair F. S. (2018). Frailty and pre-frailty in middle-aged and older adults and its association with multimorbidity and mortality: a prospective analysis of 493 737 UK Biobank participants. Lancet Public Health 3, e323–e332. Epub 2018/06/18. 10.1016/S2468-2667(18)30091-4 29908859 PMC6028743

[B13] Higa S. Ii Y. Nozawa K. Yamamoto Y. Ohwaki K. Asami Y. (2021). Relationship of annual health checkups with the subject’s subsequent behavior of cardiovascular risk management in a real-world setting in Japan: a retrospective cohort study on changes in antihypertensive drug prescription and blood pressure from 2015 to 2017. Drugs - Real World Outcomes 8, 215–225. 10.1007/s40801-020-00224-5 33598872 PMC8128959

[B14] Hirai K. Homma T. Matsunaga T. Akimoto K. Yamamoto S. Suganuma H. (2020). Usefulness of Ninjin’yoeito for chronic obstructive pulmonary disease patients with frailty. J. Altern. complementary Med. (New York, NY) 26, 750–757. Epub 2020/06/20. 10.1089/acm.2020.0083 32551796

[B15] Ito K. Kawai H. Tsuruta H. Obuchi S. (2021). Predicting incidence of long-term care insurance certification in Japan with the Kihon checklist for frailty screening tool: analysis of local government survey data. BMC Geriatr. 21, 22. Epub 2021/01/09. 10.1186/s12877-020-01968-z 33413151 PMC7792049

[B16] Lee J. S. Auyeung T. W. Leung J. Kwok T. Woo J. (2014). Transitions in frailty states among community-living older adults and their associated factors. J. Am. Med. Dir. Assoc. 15, 281–286. Epub 2014/02/19. 10.1016/j.jamda.2013.12.002 24534517

[B17] Loecker C. Schmaderer M. Zimmerman L. (2021). Frailty in young and middle-aged adults: an integrative review. J. Frailty Aging 10, 327–333. Epub 2021/09/23. 10.14283/jfa.2021.14 34549246

[B18] Miyano K. Nonaka M. Uzu M. Ohshima K. Uezono Y. (2018). Multifunctional actions of ninjinyoeito, a Japanese Kampo medicine: accumulated scientific evidence based on experiments with cells and animal models, and clinical studies. Front. Nutr. 5, 93. 10.3389/fnut.2018.00093 30349821 PMC6186795

[B19] Nakagawa Y. (1982). “Tests of the validity and reliability of the Japanese version general health questionnaire and its clinical applications,” in The theory behind understanding psychiatric and neurotic symptoms using a questionnaire and its clinical applications.

[B20] Nielsen C. R. Ahrenfeldt L. J. Jeune B. Christensen K. Lindahl-Jacobsen R. (2021). Healthy life expectancy by frailty state in europe from 2004 to 2015: findings from share. Eur. J. public health 31, 554–560. Epub 2021/02/23. 10.1093/eurpub/ckab012 33615329 PMC8485734

[B21] Ogawa K. Fujiwara Y. Yoshida H. Nishi M. Fukaya T. Kim M. (2011). The validity of the "Kihon check-list" as an index of frailty and its biomarkers and inflammatory markers in elderly people. Nihon Ronen Igakkai Zasshi 48, 545–552. Epub 2012/02/11. 10.3143/geriatrics.48.545 22323034

[B22] Puts M. T. Shekary N. Widdershoven G. Heldens J. Lips P. Deeg D. J. (2007). What does quality of life mean to older frail and non-frail community-dwelling adults in The Netherlands? Qual. life Res. Int. J. Qual. life aspects Treat. care rehabilitation 16, 263–277. Epub 2006/10/13. 10.1007/s11136-006-9121-0 17033894

[B23] Puts M. T. E. Toubasi S. Andrew M. K. Ashe M. C. Ploeg J. Atkinson E. (2017). Interventions to prevent or reduce the level of frailty in community-dwelling older adults: a scoping review of the literature and international policies. Age ageing 46, 383–392. 10.1093/ageing/afw247 28064173 PMC5405756

[B24] Rau R. Soroko E. Jasilionis D. Vaupel J. W. (2008). Continued reductions in mortality at advanced ages. Popul. Dev. Rev. 34, 747–768. 10.1111/j.1728-4457.2008.00249.x

[B25] Rizzoli R. Reginster J. Y. Arnal J. F. Bautmans I. Beaudart C. Bischoff-Ferrari H. (2013). Quality of life in sarcopenia and frailty. Calcif. tissue Int. 93, 101–120. Epub 2013/07/06. 10.1007/s00223-013-9758-y 23828275 PMC3747610

[B26] Satake S. Senda K. Hong Y. J. Miura H. Endo H. Sakurai T. (2016). Validity of the Kihon checklist for assessing frailty status. Geriatr. Gerontol. Int. 16, 709–715. Epub 2015/07/15. 10.1111/ggi.12543 26171645

[B27] Sewo Sampaio P. Y. Sampaio R. A. Yamada M. Arai H. (2016). Systematic review of the Kihon checklist: is it a reliable assessment of frailty? Geriatr. Gerontol. Int. 16, 893–902. Epub 2016/07/23. 10.1111/ggi.12833 27444395

[B28] Shinkai S. Yoshida H. Taniguchi Y. Murayama H. Nishi M. Amano H. (2016). Public health approach to preventing frailty in the community and its effect on healthy aging in Japan. Geriatr. Gerontol. Int. 16, 87–97. Suppl 1 Epub 2016/03/29. 10.1111/ggi.12726 27018287

[B29] Suzuki S. Aihara F. Shibahara M. Sakai K. (2019). Safety and effectiveness of Ninjin’yoeito: a utilization study in elderly patients. Front. Nutr. 6, 14. Epub 2019/03/16. 10.3389/fnut.2019.00014 30873411 PMC6401652

[B30] Tait R. J. French D. J. Hulse G. K. (2003). Validity and psychometric properties of the general health questionnaire-12 in young Australian adolescents. Aust. N. Z. J. psychiatry 37, 374–381. Epub 2003/06/05. 10.1046/j.1440-1614.2003.01133.x 12780478

[B31] Takayama S. Arita R. Ohsawa M. Kikuchi A. Yasui H. Makino T. (2019). Perspectives on the use of Ninjin’yoeito in modern medicine: a review of randomized controlled trials. Evidence-Based Complementary Altern. Med. 2019, 1–15. 10.1155/2019/9590260 PMC6745181 31565066

[B32] Trevisan C. Veronese N. Maggi S. Baggio G. Toffanello E. D. Zambon S. (2017). Factors influencing transitions between frailty states in elderly adults: the progetto veneto anziani longitudinal study. J. Am. Geriatr. Soc. 65, 179–184. Epub 2016/11/20. 10.1111/jgs.14515 27861714

[B33] Tsutsui T. Muramatsu N. (2005). Care-needs certification in the long-term care insurance system of Japan. J. Am. Geriatr. Soc. 53, 522–527. Epub 2005/03/04. 10.1111/j.1532-5415.2005.53175.x 15743300

[B34] Tsutsui T. Muramatsu N. (2007). Japan’s universal long-term care system reform of 2005: containing costs and realizing a vision. J. Am. Geriatr. Soc. 55, 1458–1463. Epub 2007/09/05. 10.1111/j.1532-5415.2007.01281.x 17767690

[B35] United Nations (2023). World population aging 2023. New York, USA: Desa Publications.

[B36] Uto N. S. Amitani H. Atobe Y. Sameshima Y. Sakaki M. Rokot N. (2018). Herbal medicine Ninjin’yoeito in the treatment of sarcopenia and frailty. Front. Nutr. 5, 126. 10.3389/fnut.2018.00126 30619872 PMC6299011

[B37] Williams F. R. Berzigotti A. Lord J. M. Lai J. C. Armstrong M. J. (2019). Review article: impact of exercise on physical frailty in patients with chronic liver disease. Alimentary Pharmacol. Ther. 50, 988–1000. Epub 2019/09/11. 10.1111/apt.15491 31502264

[B38] Zhang L. Clark T. Gopalasingam G. Neely G. G. Herzog H. (2021). Ninjin’yoeito modulates feeding and activity under negative energy balance conditions via the npy system. Neuropeptides 87, 102149. Epub 2021/04/22. 10.1016/j.npep.2021.102149 33882337

